# Correlation-centred variable selection of a gene expression signature to predict breast cancer metastasis

**DOI:** 10.1038/s41598-020-64870-z

**Published:** 2020-05-13

**Authors:** Shiori Hikichi, Masahiro Sugimoto, Masaru Tomita

**Affiliations:** 1 Graduate School of Media and Governance, Keio University, Fujisawa, Kanagawa, 252-8520 Japan; 2 Research Fellow of Japan Society for the Promotion of Science, Chiyoda, Tokyo, 102-0083 Japan; 3 Institute for Advanced Biosciences, Keio University, Tsuruoka, Yamagata, 997-0811 Japan; 4 Research and Development Center for Minimally Invasive Therapies, Institute of Medical Science, Tokyo Medical University, Shinjuku, Tokyo, 160-0022 Japan

**Keywords:** Gene ontology, Predictive markers

## Abstract

Predictions of distant cancer metastasis based on gene signatures are studied intensively to realise precise diagnosis and treatments. Gene selection i.e. feature selection is a cornerstone to both establish accurate predictions and understand underlying pathologies. Here, we developed a simple but robust feature selection method using a correlation-centred approach to select minimal gene sets that have both high predictive and generalisation abilities. A multiple logistic regression model was used to predict 5-year metastases of patients with breast cancer. Gene expression data obtained from tumour samples of lymph node-negative breast cancer patients were randomly split into training and validation data. Our method selected 12 genes using training data and this showed a higher area under the receiver operating characteristic curve of 0.730 compared with 0.579 yielded by previously reported 76 genes. The signature with the predictive model was validated in an independent dataset, and its higher generalization ability was observed. Gene ontology analyses revealed that our method consistently selected genes with identical functions which frequently selected by the 76 genes. Taken together, our method identifies fewer gene sets bearing high predictive abilities, which would be versatile and applicable to predict other factors such as the outcomes of medical treatments and prognoses of other cancer types.

## Introduction

Breast cancer is the most common cancer in women worldwide^1^. It is a heterogeneous disease that causes difficulties for its diagnosis and treatment. Thus, multiple clinicopathological factors, such as the tumour size and lymph node metastasis, and established biomarkers, such as the estrogen receptor (ER), human epidermal growth factor receptor 2 (HER2), and progesterone receptor, have been used to characterise specific features of breast cancer, including its subtypes. To realise more accurate characterisation of breast cancer, gene expression has been introduced to understand molecular-level pathology and prediction of treatment outcomes of breast cancer, which would contribute to the design of treatments^2,3^. Currently, MammaPrint^4^, Oncotype DX^5^, and MapQuant Dx^6^ are commercially available diagnostic tests to accurately estimate the recurrence of breast cancer. These tests employ relatively large numbers of genes, that is, 70, 21, and 97, respectively. Gene expression is also used for metastasis prediction and subtype identification of breast cancers^7–9^. The use of gene expression in combination with known biomarkers and clinicopathological factors is considered as a critical issue for precision medicine.

There are three processes in the establishment of a prediction model using microarray data: (1) feature selection, (2) development of a mathematical model, and (3) validation of the developed model. The purpose of feature selection is to identify a minimal predictive gene set by removing redundant and irrelevant features^10–12^. This process is important because the subsequent analyses depend on the selected genes. The prediction performance of a mathematical model is changeable depending on the selected genes^13^. There are several feature selection techniques including feature ranking as a well-established approach^10^, and unconditional mixture modelling, information gain ranking, and Markov blanket filtering as more modern approaches^14^. Variance inflation factor (VIF) is an index to assess the dependence of features^15^. These methods were designed to identify a minimal feature set by reducing multicollinearity. However, more accurate and noise-robust methods are needed.

Mathematical prediction models are classified into statistical and machine-learning approaches. As statistical approaches, multivariable logistic regression has been used for binary outcomes such as metastasis^16^. As machine-learning methods, artificial neural networks, Bayesian networks, support vector machines (SVMs), and decision trees (DTs) are typical methods for binary outcomes^17^, and machine learning-based analyses have been applied to the prediction of cancer prognosis^17–19^. Combining multiple methods has also been reported, such as ReliefF-GA-ANFIS^18^. The prediction accuracy of a prediction model is dependent on data. However, a generalised combinatorial approach should be developed to choose both feature selection and a prediction model.

As a general problem, both feature selection and model development depend on datasets and various conditions, especially user-defined parameters. For example, random splitting of datasets into training and validation data is a common approach to confirm the generalisation ability of the developed model. However, many studies evaluate the developed model using only one split condition, which might alter the prediction accuracies. To assess the generalisation ability of the developed model, rigorous evaluations should be performed using various conditions.

This study aimed to develop a protocol to realise accurate prediction by selecting minimal gene sets. As a case study, we used datasets to predict metastasis of breast cancer by applying a multiple logistic regression (MLR) model with a 76 gene signature^20^. We also employed MLR while addressing the design of a novel feature selection. Using various split conditions, our feature selection approach successfully identified fewer but more accurate gene signatures. External validation also proved the robustness of our approach.

## Methods

### Data collection and pre-processing

**Figure 1:**
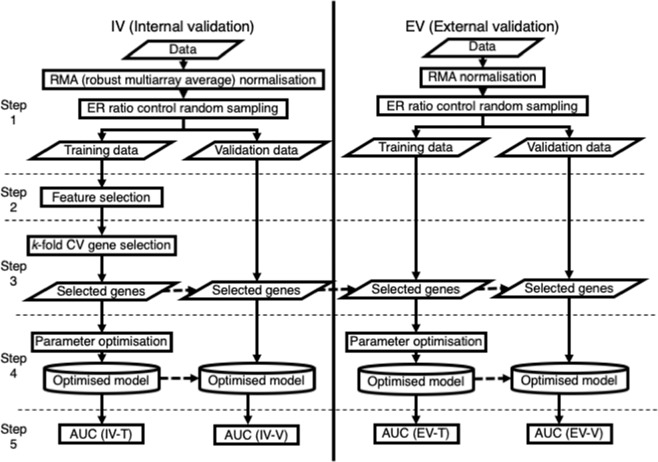
Flowchart for identification of a gene expression signature. Whole processes are described in five steps including pre-processing, feature selection, gene count selection, training, and validation of the prediction model. For internal validation using GSE2034, a set of gene signatures was selected and a prediction model was developed. For external validation using independent datasets (TRANSBIG), the identical gene set selected using GSE2034 was used for the features of the model. However, the parameters of the model are optimized using training data of TRANSBIG and the developed model is validated the validation data of TRANSBIG.

A flowchart to identify genes associated with breast cancer metastasis is illustrated in Fig. 1 and Figure S1. To demonstrate the applicability of the proposed gene selection and evaluate its consistency, we used three independent Affymetrix Human U133a GeneChip^21^ datasets. The gene expression data were obtained from tumour samples of lymph node-negative breast cancer patients who were not treated by systemic therapy after surgery. In total, datasets of 286 and 302 subjects were retrieved from GSE2034^20^ and TRANSBIG including GSE6532^22^ and GSE7390^23^, respectively, through the NCBI Gene Expression Omnibus^24^. Various subtypes were included in the datasets. For example, the data in GSE2034 included subjects with lymph node- and ER + data, and GSE6532 included ER + data. All microarray data were normalised by the robust multichip average (RMA) algorithm with the default option^21,25^.

### Identification of predictive gene sets

Each microarray dataset was randomly separated into training and validation datasets. The ratio of samples included in the training and validation datasets was approximately 2:1. To eliminate the effects of the ER on our model predictions, we divided the samples according to the ER status. Gene selection and training the mathematical model using the selected genes were performed using the training data. The generalisation ability of the developed model was evaluated using the validation data.

Each gene was individually evaluated to select genes showing a difference between samples with or without 5-year metastasis. First, genes showing a significant difference using conventional statistical analysis (Student’s t-test) were selected. Second, the remaining genes were ranked based on the margin between the two groups, and we selected only highly ranked genes by support vector machine recursive feature elimination (SVM-RFE)^26^. Various subsets including a different number of genes were compared by the area under the receiver operating characteristic curve (AUC). Of the remaining genes, VIF was used to select minimal genes that were independent such as low positive correlations among the genes.

### Optimisation of the gene count

The gene count should be optimised in gene sets selected by feature selection procedures, which is the number of genes used for the prediction method. One simple method is to determine the gene count by tuning the options of feature selection. However, in this study, we directly evaluated the accuracy of the model with various gene counts, such as the use of subset genes included in the gene sets selected by the feature selection procedure. In general, a prediction model using a larger number of genes tends to show higher prediction performance for the given training data, but a lower generalisation ability for validation data. To eliminate this over-fit tendency, we performed *k*-fold cross-validation (CV), and AUC was used to evaluate both sensitivity and specificity. The AUC values and gene count were calculated by CV using training data. The differential values of AUC were calculated based on the change in the differential value.

### Model development and validation

Gene selection was conducted using GSE2034, which is defined as internal validation here. GSE2034 was used as model development and internal validation (IV), i.e. the data was randomly split into internal training (IV-T) and validation (IV-V) datasets. To evaluate the generalization ability of selected genes, we used the TRANSBIG dataset including GSE7390 and GSE6532 as external validation datasets (EV). Here, two external validation tests were conducted. First, only the generalization ability of the gene signature was evaluated. The TRANSBIG dataset was randomly split into two datasets as external training (EV-T) and external validation (EV-V). The models were developed using the EV-T with the genes selected by GSE2034, and the developed model was evaluated using the EV-V. This TRANSBIG dataset was not used for the selection of genes (Exp. 1, Fig. 1). Secondly, the generalization ability of both the developed model and the gene signature was evaluated. The model development and the selected genes in Exp. 1 was conducted using IV-T, this model was evaluated using whole TRANBIG datasets (EV). To eliminate the experimental bias, intensities of the genes of each dataset were normalized into Z-score. Here, gene selection and model development were not conducted using TRANSBIG (Exp. 2, Figure S1).

Here, we used the following parameters. To independently evaluate genes showing a difference between the two groups with or without 5-year metastasis, genes showing small intensities (median intensity: <5 in a group) were eliminated, and genes with *P* ≤ 0.005 (Student’s t-test) had remained. SVM-RFE was conducted using a linear kernel with the default option. The number of genes selected by SVM-RFE changed from 2, 7, 12, and 102. Generally, VIF exceeding 10 is an indicator of requiring serious multicollinearity consideration. As a threshold of multi-linearity, VIF < 10 was used. The differential values were sorted, and the value in the top 25% was used as the threshold to determine the gene count. All programs were implemented using Python (version 3.7.0), R (version 3.3.0), Numpy (version 1.18.2), Scipy (version 1.4.1), Matplotlib (version 3.2.1), seaborn (version 0.10.0), scikit-learn (version 0.22.2), PypeR (version 1.1.2) and JMP (version 14.0.0). These programs are distributed upon request. DAVID Bioinformatics Resources 6.8 was used for gene ontology analyses.

## Results

### Prediction accuracy of gene signatures

To evaluate our proposed methods, we developed and validated a prediction model using the same data employed by Wang *et al*.^20^. They used a dataset named GSE2034 and (1) randomly split the data into training and validation sets, (2) explored a 76 gene signature and trained the MLR model using the training data, and (3) evaluated the model using the validation data. We also used these data and split them into two datasets: training and validation datasets. Of the 22283 genes, conventional statistical analyses selected 208 genes showing a significant difference between the two groups with or without 5-year metastasis. Subsequently, 12 genes were selected by VIF.

**Figure 2:**
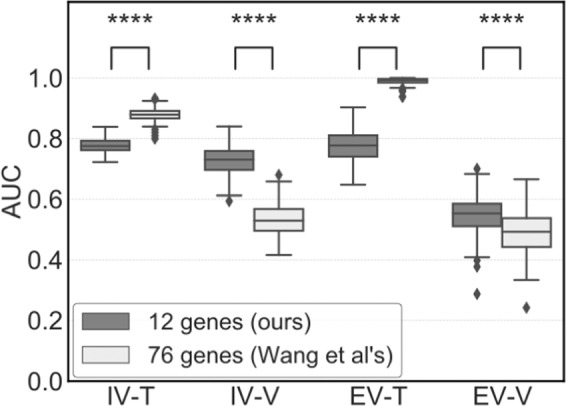
Prediction performance of the two gene sets (Exp. 1). AUC values to discriminate between patients with or without metastasis. Training (IV-T) and validation datasets (IV-V) were generated by 200 random splits of (**a)** GSE2034 as the internal validation dataset and (**b)** the TRANSBIG dataset including GSE6532 and GSE7390 as the external validation dataset. This dataset was also randomly split into training (EV-T) and validation datasets (EV-V). The difference in AUC values was evaluated by Mann-Whitney tests. The values of the median, and 1^st^ and 3^rd^ quartiles are plotted by horizontal bars. Data outside more than one and a half times the length of the boxplot (3^rd^ quartile minus 1^st^ quartile; interquartile range) were plotted. Significant differences are shown (*P < 0.05, **P < 0.01, ***P < 0.005, and ****P < 0.001).

For rigorous assessment, we performed 200 random splits of the data into training and validation datasets using different random values. In the internal validation, the AUC values of the training data (IV-T) were similar, where median values were 0.775 and 0.869 for 12 and 76 genes, respectively. However, these values of the validation data (IV-V) were significantly different (*P* < 0.001), where median values were 0.730 and 0.529 for 12 and 76 genes, respectively (Fig. 2).

### External evaluation

The generalisation abilities of the gene signatures yielded by GSE2034 were evaluated using an independent dataset (TRANSBIG). At the Exp.1, the TRANSIBIG dataset was randomly split into training and validation datasets as internal validations (IV-T and IV-V). The genes used for the MLR models was not selected by this data while coefficients of the MLR model was optimized using the IV-T and evaluated these models using IV-V. This process was repeated 200 times with different random values. In the external validation, the MLR models were developed using EV-T and evaluated EV-V. The median AUC values of our 12 and 76 genes were 0.553 and 0.493, respectively, which were significantly different (*P* < 0.001) (Fig. 2).

**Figure 3:**
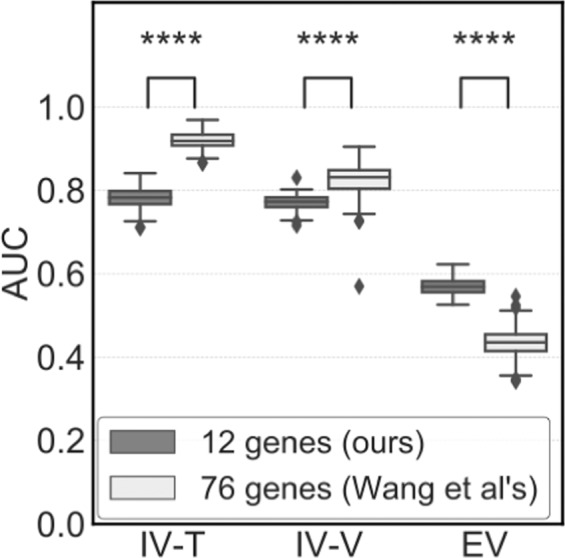
Prediction performance of MLR models of 12 and 76 genes (Exp. 2). AUC values to discriminate between patients with or without metastasis. As model optimisation for independent datasets, the set of gene signatures selected in the process of *k*-fold CV gene selection in Fig. 1 was normalised using Z-score. Training and validation datasets were generated by 200 random splits of (**a)** GSE2034 as the internal validation dataset (IV-T, IV-V) and (**b)** the TRANSBIG dataset including GSE6532 and GSE7390 as the external validation dataset (EV). The difference in AUC values was evaluated by Mann-Whitney tests. The values of the median, and 1^st^ and 3^rd^ quartiles are plotted by horizontal bars. Data outside more than one and a half times the length of the boxplot (3^rd^ quartile minus 1^st^ quartile; interquartile range) were plotted. Significant differences are shown (*P < 0.05, **P < 0.01, ***P < 0.005, and ****P < 0.001).

As the Exp. 2, both of the MLR model and selected genes were evaluated. The MLR model was developed using IV-T with 12 genes selected in Exp.1. This model was evaluated using EV-V as an external validation. The median AUC values of our model with 12 genes and the model of Wang *et al*. were 0.569 and 0.436, respectively (Fig. 3).

### Comparison of the gene functions of selected gene subsets

**Figure 4:**
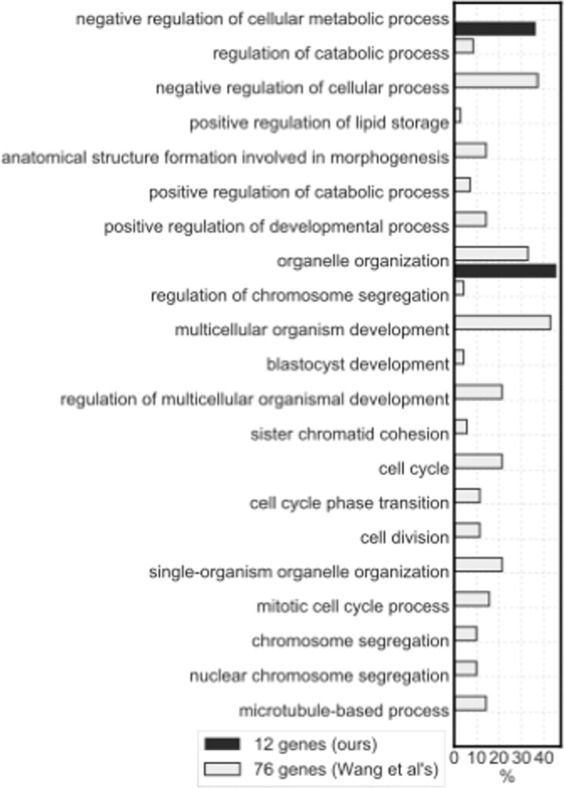
Gene ontology included in the genes selected from the public microarray dataset and gene sets reported by Wang *et al*. The percentage of each functional annotation indicates the appearance frequency. To normalise the number of each functional annotation, the ratio of the number of gene ontology terms (GOTERMs) to the total number of selected genes is shown. The GOTERM category focuses on the third threshold of the biological process in DAVID Bioinformatics Resources 6.8.

To evaluate the gene functions of gene signatures, we assigned gene ontology terms (GOTERMs) to each gene by DAVID analysis. Figure 4 shows the comparison of pathways included in our identified gene set and Wang *et al*.’s gene set. The proportion of the number of gene functions attributed to each category to the total number of genes was visualised. One category was consistently included in both 12 and 76 gene signatures, namely organelle organization. Organelle organization had the largest number of genes in both signatures. Table S2 shows the prediction performance, that is, AUC and odds ratio, of the 12 genes selected by our method.

### Effect of each option to generate a gene signature and prediction model

Because various options for prediction model development potentially affect the gene count, we investigated the relationship between these options and gene counts.

**Figure 5:**
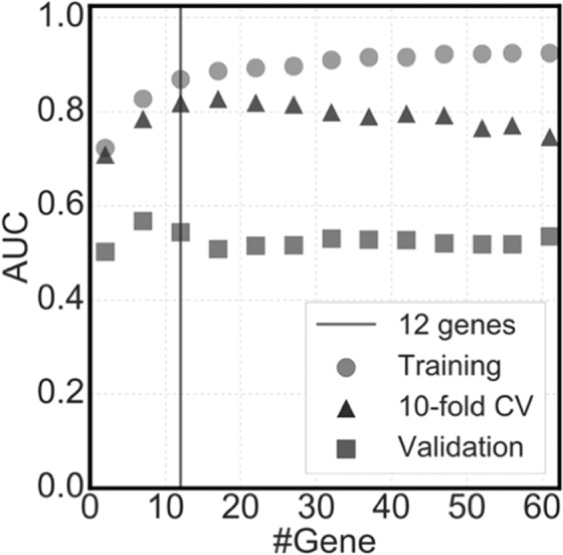
Prediction performance of the three datasets focusing on the number of genes. The accuracies of prediction models (AUC) were calculated after random sampling (training and validation datasets) and *k*-fold cross-validation (*k*-fold CV dataset). For a suitable gene count, 12 genes were selected with the list of AUCs of *k*-fold cross-validation. #Gene indicates the number of genes.

First, we analysed the effect of the number of genes on the prediction performance (Fig. 5). The relationship between the gene count and model accuracy was depicted, which were yielded using whole training data, CV using training data, and validation data. As expected, the AUC values of training data increased monotonically along with gene counts, while those of CV became almost constant even though the gene count increased. The AUC values of validation data also became almost constant at a gene count of >20 and slightly decreased at a gene count of >50.

**Figure 6:**
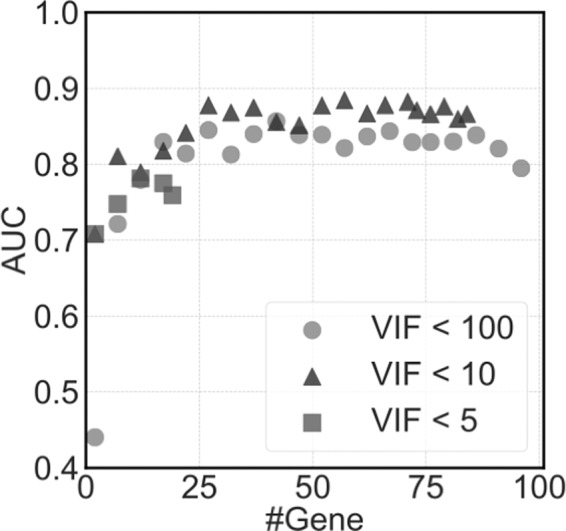
Prediction performance of the three parameters in the variance inflation factor-based gene selection. The accuracies of prediction models (AUC) was calculated with three thresholds of the variance inflation factor (VIF): the normal threshold of multicollinearity (VIF < 10), the weaker threshold (VIF < 100), which selects more genes than the normal threshold, and the stricter threshold of multicollinearity (VIF < 5) that selects fewer genes than the normal threshold.

To select the gene count, VIF criteria were used to eliminate multicollinearity among genes. Here, we compared different VIF criteria, <5, <10, and <100. The relationship between the gene count and AUC values of *k*-fold CV is depicted in Fig. 6. Using stricter thresholds of <5 and <10, 20 and 85 genes were selected at maximum, respectively. The AUC values of any VIF threshold showed similar values at a small gene count of <20, while those of VIF at <10 showed a slightly higher trend in most cases compared with a VIF of <100.

GSE2034 data were randomly split into training and validation data. The sample ratio between these datasets was approximately 2:1, according to the condition employed by Wang *et al*.^20^. To investigate the effect of this ratio on the prediction accuracies, we compared various split ratios, 1:1, 2:1, and 3:1, for training and validation data (Figure S2). At lower gene counts of <25, the AUC values were almost the same among these split ratios. These trends were also observed between 1:1 and 2:1 at larger gene counts of >70. Furthermore, in the middle range of gene counts, the AUC values were different among them. The accuracies of the model were also compared based on a 95% confidential interval (CI) (Table S1). The 95% CI is the index to indicate the stability of prediction accuracy, and the narrowest range of 95% CI is the best split ratio.

The results of CV are also dependent on the *k*-value that also affects gene counts. Thus, we compared *k* = 3, *k* = 5, and *k* = 10, and explored the relationship between gene counts and AUC values of *k*-fold CV using training data (Figure S3). The AUC values for *k* = 5 and *k* = 10 showed similar trends regardless of the gene count, whereas those of *k* = 3 were slightly decreased at gene counts of>30.

Next, we investigated the effect of the prediction model of the gene count. In addition to MLR, we used linear SVM and random forest (RF), which are commonly used classification methods, and depicted the relationship between gene counts and their AUC values of 10-fold CV using training data (Figure S4). AUC of the MLR model almost gradually increased along with gene counts and became almost constant at gene counts of >30. Moreover, AUC values of linear SVM and RF more strongly depended on the gene counts compared with MLR, where these values sharply increased at smaller gene counts and decreased gradually at larger gene counts.

The relationships between the model and other variables in the TRANSBIG dataset, such as the ER status, tumour size, and HER2, were analysed (Figure S5). The effects of these parameters on the probability of metastasis within 5 years predicted by our model showed no significant difference between non-metastasis and metastasis subjects, indicating the independence of their relationship.

## Discussion

In this study, we addressed the feature selection of gene expression profiles. Many feature selection methods have been developed to remove redundant and irrelevant features from high dimensional microarray data^10–12^. To use multiple genes for prediction models such as MLR, the multicollinearity of these genes should be carefully dealt with because this feature frequently deteriorates the generalisation ability of the model. Therefore, we developed a simple but effective feature selection method to minimise the number of genes in identical gene sets by eliminating multicollinearity while showing high accuracy and generalisation abilities.

Various approaches are available for gene selection by eliminating collinearity. The data-dependent centred method centres two variables around their means to avoid creating multicollinearity^15^. Clustering, such as *k*-means, can also be used as a feature selection method by selecting representative features among clusters. Each cluster includes correlated features, and the combination of representative features among clusters is expected to have low collinearity. Feature subset selection can also be used for division into strong and weak relevant features with DTs and Naive-Bayes. However, these feature selection algorithms are not enough for application to robust data such as microarray data including multicollinearity, because of the trade-off between the quality of the estimates and the search size^27^. In this study, we used SVM and VIF to rank the features and evaluate multicollinearity, which revealed independent gene sets showing high discrimination abilities.

A prediction model should bear robustness against various conditions. Gene count, which fluctuates depending on the gene selection procedure, is one of the possible factors that affect the prediction ability of a model. RF has been frequently used to predict the outcomes of breast cancer treatments, such as prediction of a pathological complete response after adjuvant therapy^19^ and risk assessment of 10-year relapse-free patients with node-negative, ER+ , and HER2- breast cancer^28^. RF itself evaluates the importance of the variable in the developed model and therefore can be used for feature selection^29^. However, our numerical experiments revealed that the prediction performance of RF strongly depended on the input variables (Figure S4), indicating that feature selection before the RF should be designed carefully. SVM can be used for feature selection^30^. An SVM has been used for breast cancer diagnosis^31^ and used with feature selection, such as the use of *k*-mean clustering, before training of a model^32^. Our results also showed high sensitivity of the SVM model against the number of genes. Taken together, feature selection should be evaluated with the prediction ability of a mathematical model.

Wang *et al*. used GSE2034 and randomly split datasets into training and validation datasets, developed an MLR model using 76 genes by employing training data, and evaluated the generalisation ability of the model using validation datasets, which resulted in AUC = 0.694 using validation datasets^20^. However, we repeated the identical procedure with different random values, resulting in lower generalisation abilities using their signature. Also, the external validation showed no predictive abilities at AUC < 0.5 (Exp. 1). Thus, rigorous internal validation should be conducted to optimise gene signatures before validation using external datasets. We also evaluated not only the gene signature but also the MLR model with gene signatures (Exp. 2). In this validation test, the model of Wang *et al*. yielded AUC = 0.404 while our model yielded AUC = 0.560 (median values in 200 trials) using external validation data (Fig. 3). This test also revealed the Wang *et al.’*s method showed less generalization ability.

Wang *et al*. used the ER status to split datasets into training and internal validation data. Therefore, the effect of ER on the prediction accuracy was not analysed^20^. In this study, we also split the training data (GSE2034) considering the ER status. We analysed the relationships among the model and other variables in the TRANSBIG dataset, such as the ER status, tumour size, and HER2 (Figure S5), and found a small effect of these variables on the prediction accuracies.

Several limitations need to be acknowledged in this study. We employed commonly used feature selection methods and multi-linearity evaluation criteria, SVM-RFE and VIF. However, there are various other methods and criteria available. Other combinations should also be compared. As a case study, we addressed the prediction of breast cancer metastasis. However, our approach should be validated to predict other outcomes to obtain a more general conclusion. Here, we assessed the effect of various options of gene signature selection. However, we still used a certain threshold for each procedure and did not evaluate multiple parameters simultaneously. Although we analysed the relationships among microarray data and metastasis probability, other various factors, such as smoking, were not incorporated into our prediction, because these factors were not recorded in the employed data. A predictive tool based on multivariable analysis using these factors and microarray data has the potential to improve the accuracy of prediction. We will provide all source code upon request to contribute to the development of such tools. Here, we evaluated the selected genes and confirmed the higher generalization ability using independent datasets compared with the method of Wang *et al*.^20^ (Exp. 1). The model with gene signatures also evaluated using independent datasets and this test also revealed that the less generalization ability of Wang *et al*.^20^ (Exp. 2). These experiments revealed the larger number of genes was not robust for external datasets.

We have developed novel variable selection to improve the precision accuracy of cancer metastasis using fewer genes regardless of the input microarray datasets. This study confirmed consistency using robust feature selection that has high repeatability and robustness. Our proposed approach may contribute to medical treatments for breast cancer patients.

## Supplementary information

Supplementary Information.
