# Supplementary material for: Correlation-centred variable selection of a gene expression signature to predict breast cancer metastasis
Source: Sci Rep. 2020 May 13;10:7923. doi: 10.1038/s41598-020-64870-z (PMC7220948; doi:10.1038/s41598-020-64870-z)
Supplement: Supplementary file 1 — Supplementary Information. [file 41598_2020_64870_MOESM1_ESM.pdf]

Correlation-centred variable selection of a gene expression signature to predict breast cancer metastasis

Shiori Hikichi[1,2], Masahiro Sugimoto[3,4*] and Masaru Tomita[1,3]

[1]Graduate School of Media and Governance, Keio University, Fujisawa 252-8520, Japan
[2]Research Fellow of Japan Society for the Promotion of Science, Tokyo 102-0083, Japan
[3]Institute for Advanced Biosciences, Keio University, Tsuruoka, Yamagata 997-0811, Japan
[4]Research and Development Center for Minimally Invasive Therapies, Institute of Medical Science, Tokyo Medical University, Shinjuku, Tokyo 160-0022, Japan.

*Email: mshrsgmt@gmail.com, mshrsgmt@tokyo-med.ac.jp

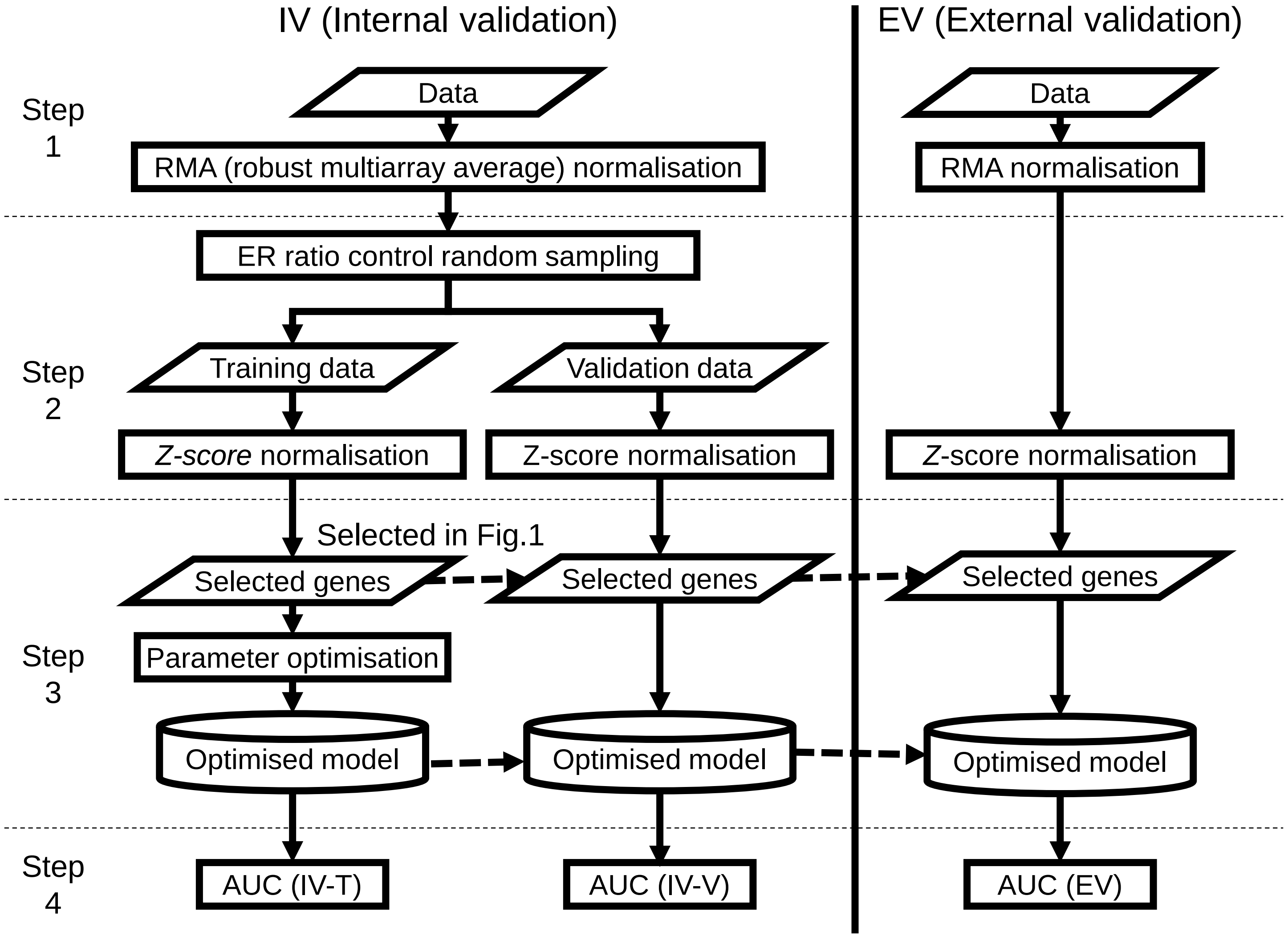

Figure S1. Flowchart for model optimisation using an independent dataset. Whole processes are described in four steps including normalisation, random sampling, training, and validation of the prediction model. The gene signatures selected in Figure 1 are used. Each gene was normalised by Z-Score to eliminate the bias of the average and the deviations of each dataset. The GSE2034 and TRANSBIG were used for internal and external validations, respectively. The model is developed using the IV-T and validated by IV-V of internal validation (IV). The model is validated using external validation (EV) data.

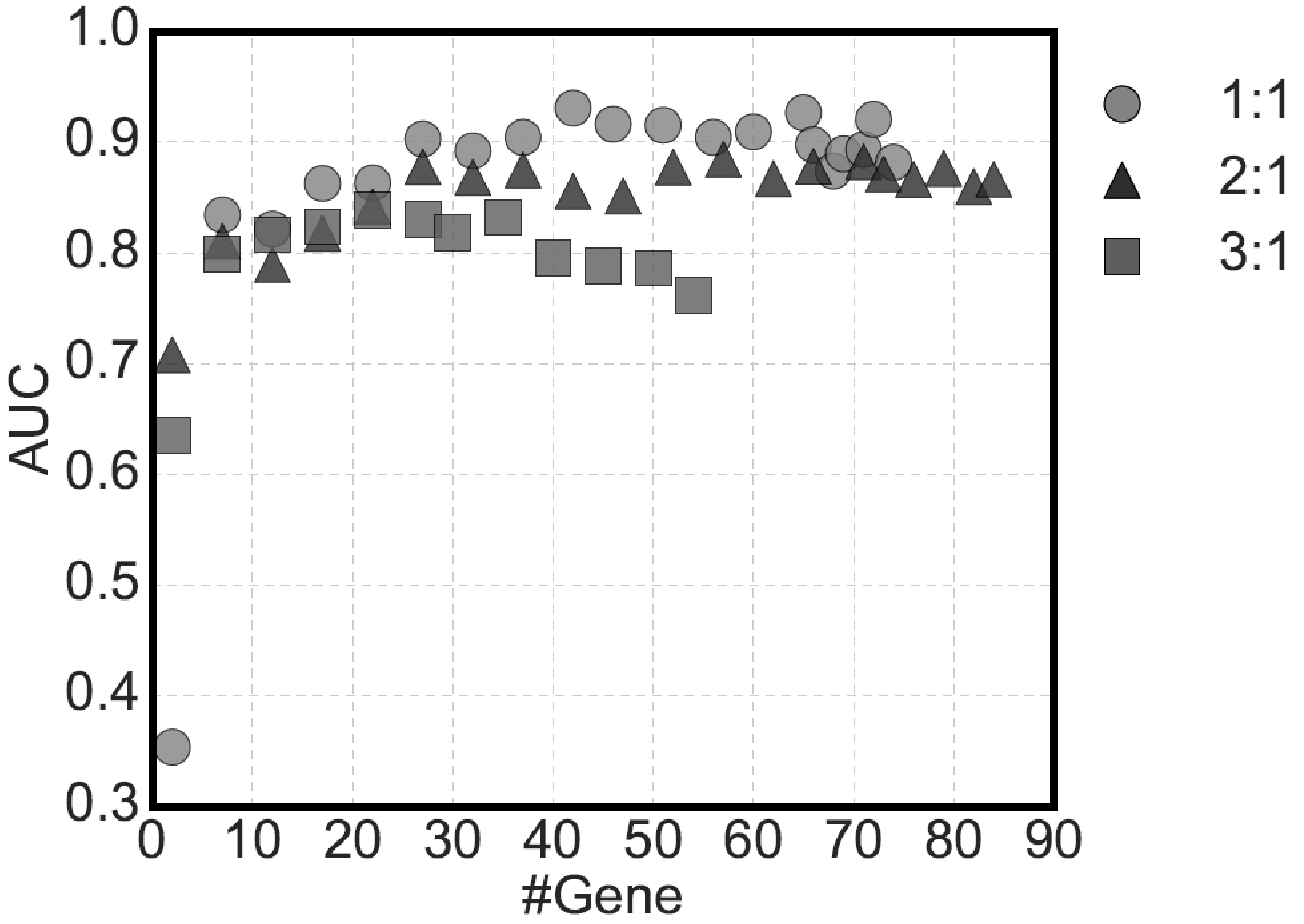

Figure S2. Prediction performance of the three split ratio in random sampling. The accuracies of prediction models (AUC) were calculated after dividing the input data into training and validation datasets by several ratios: 1/2 and 1/2 training and validation datasets, 2/3 and 1/3 training and validation datasets, and 3/4 and 1/4 training and validation datasets.

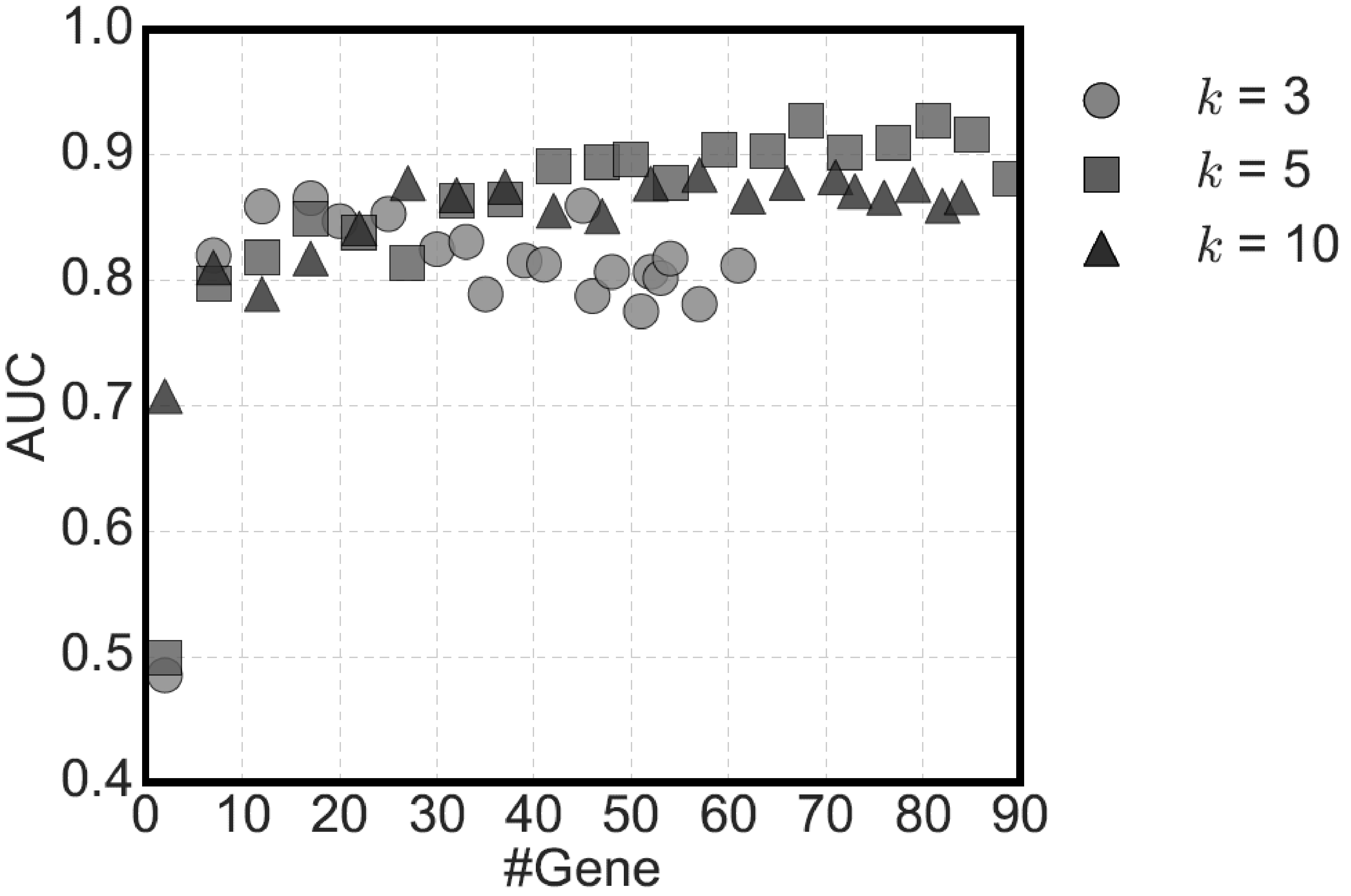

Figure S3. Prediction performance of the three parameters in *k*-fold CV. The accuracies of prediction models (AUC) were calculated by several parameters *k* of *k*-fold CV in the step of decision of a suitable gene count: *k* = 3, *k* = 5, and *k* = 10.

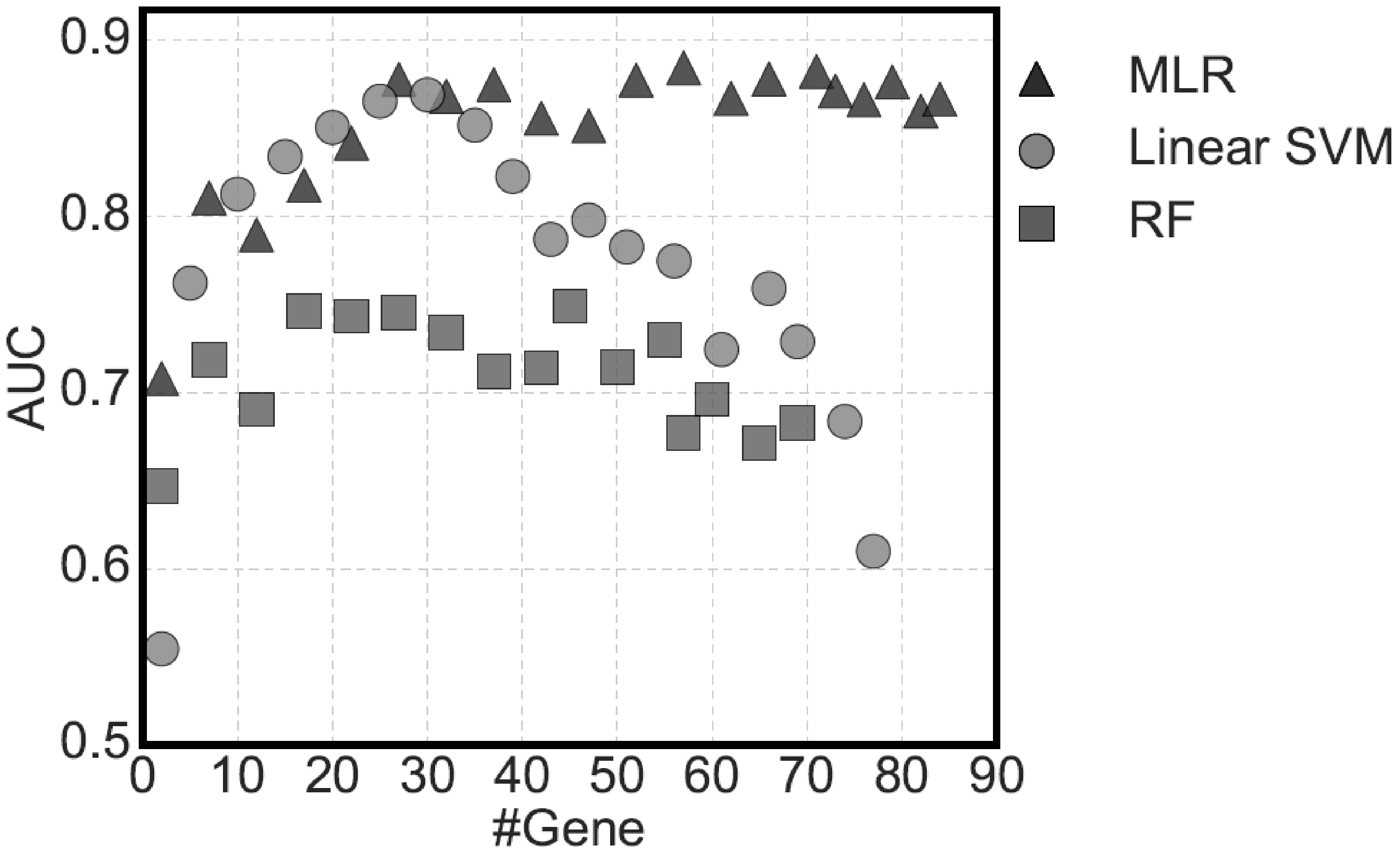

Figure S4. Prediction performance of the three prediction models. The accuracies of prediction models (AUC) were calculated by the three prediction models: linear SVM, multivariate logistic regression (MLR), and random forest (RF).

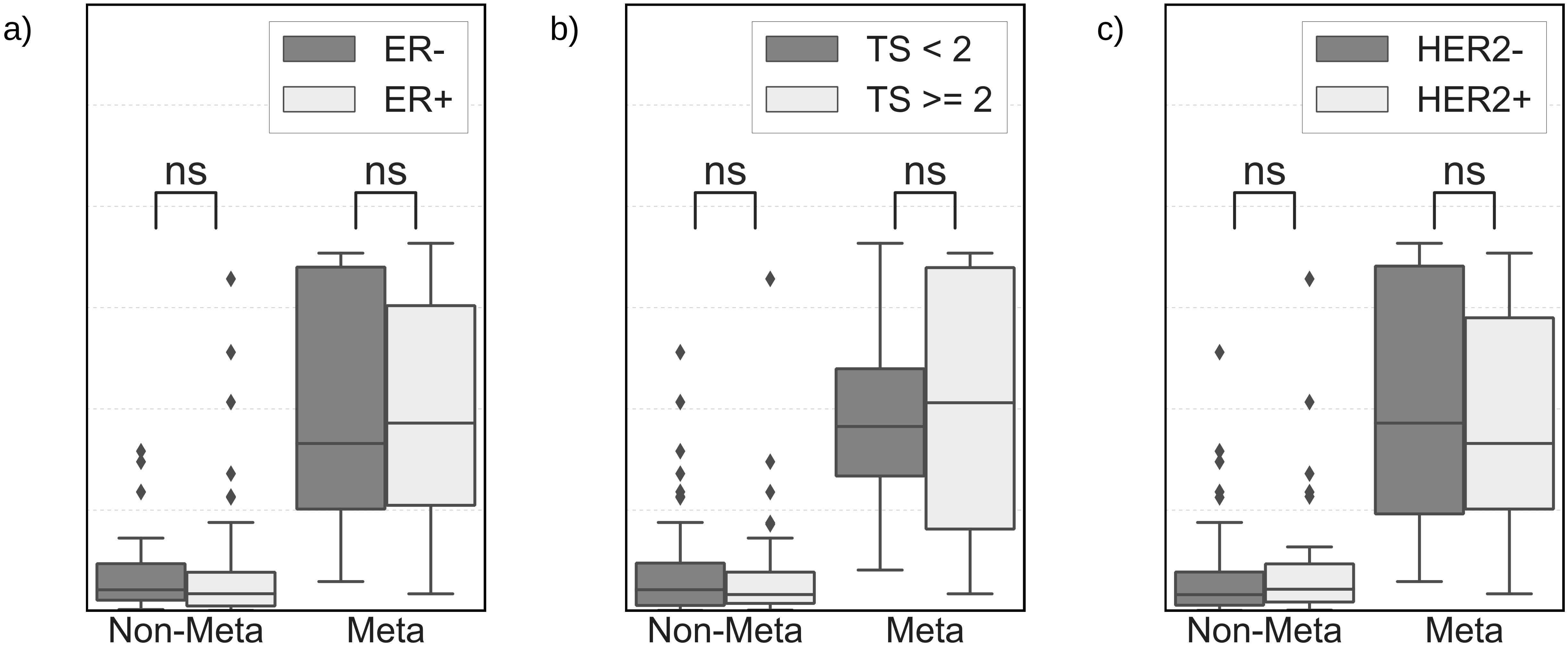

Figure S5. Parameter effects on probability of metastasis in the TRANSBIG dataset.
a) ER status, **b)** tumour size, and **c)** HER2 status. Horizontal bars on the box indicate the median, and 1st and 3rd quartiles. Outliers (≥1.5 times of 3rd–1st quartiles) are plotted as dots. Significant differences are shown (Mann-Whitney tests, ns $P \geq 0.05$, *$P < 0.05$, **$P < 0.01$, ***$P < 0.005$, and ****$P < 0.001$). Meta, Non-Metam, and TS indicate metastasis, non-metastasis, and tumour size (cm), respectively.

Table S1. Prediction model stability of the three split ratio in random sampling.

Ratio of Data (training:validation)	Median of Accuracy after CV	95% Confidence Interval of Accuracy after CV
1:1	70.330	(43.171, 97.488)
2:1	68.421	(45.762, 91.081)
3:1	72.500	(42.169, 102.831)

Median of accuracy after CV and the 95% confidence interval of accuracy after CV were calculated after dividing the target data into training and validation datasets by several ratios: 1/2 and 1/2 training and validation datasets, 2/3 and 1/3 training and validation datasets, and 3/4 and 1/4 training and validation datasets.

Table S2. Prediction performance of the genes selected from the public microarray dataset.

Gene	Affymetrix probe ID	Gene name	Internal validation			External validation		
			AUC	Odds ratio	P-value	AUC	Odds ratio	P-value
1	213826_s_at	H3 histone family member 3A (H3F3A)	0.627	0.316	0.0006	0.505	0.997	0.93
2	213381_at	V-set and transmembrane domain containing 4 (VSTM4)	0.524	1.22	0.52	0.501	0.981	0.98
3	216246_at	-	0.614	0.439	0.002	0.570	0.847	0.25
4	217139_at	Voltage dependent anion channel 1 (VDAC1)	0.568	0.180	0.066	0.553	0.334	0.38
5	202028_s_at	Ribosomal protein L38 (RPL38)	0.606	0.505	0.0042	0.539	0.881	0.52
6	212484_at	Family with sequence similarity 89 member B (FAM89B)	0.599	0.339	0.0074	0.591	0.521	0.13
7	218250_s_at	CCR4-NOT transcription complex subunit 7 (CNOT7)	0.596	1.61	0.0095	0.600	2.18	0.10
8	214045_at	Lipoic acid synthetase (LIAS)	0.628	0.199	0.0005	0.610	3.13	0.070
9	201178_at	F-box protein 7 (FBXO7)	0.600	3.48	0.0069	0.541	1.35	0.50
10	78383_at	-	0.572	3.20	0.051	0.549	1.91	0.42
11	207555_s_at	Thromboxane A2 receptor (TBXA2R)	0.636	7.41	0.0002	0.594	3.06	0.12
12	208205_at	Protocadherin alpha 9 (PCDHA9)	0.566	0.269	0.073	0.593	0.265	0.13

Median of AUC in 200 trials, odds ratio, and P-value regarding metastasis within five years were calculated using the dataset reported by Wang *et al*. for internal validation and the public microarray TRANSBIG dataset for external validation. Significant differences are shown (*$P < 0.05$, **$P < 0.01$, ***$P < 0.005$).
